# High-quality human preimplantation embryos actively influence endometrial stromal cell migration

**DOI:** 10.1007/s10815-017-1107-z

**Published:** 2017-12-28

**Authors:** R. P. Berkhout, C. B. Lambalk, J. Huirne, V. Mijatovic, S. Repping, G. Hamer, S. Mastenbroek

**Affiliations:** 1 Center for Reproductive Medicine, Academic Medical Center, University of Amsterdam, 1105 AZ Amsterdam, The Netherlands; 2 Department of Obstetrics and Gynecology, IVF Center, VU University Medical Center, 1081 JC Amsterdam, The Netherlands

**Keywords:** Implantation, Endometrium, Embryo, Cross-talk, Decidualization

## Abstract

**Purpose:**

The purpose of this paper is to study whether human preimplantation embryos regulate endometrial stromal cell (hESC) migration.

**Methods:**

Primary hESCs were isolated from fertile patients undergoing hysterectomy for benign conditions (uterine scar niche *n* = 3, dysmenorrhea *n* = 2; no hormonal treatment). Migration and proliferation assays were performed by culturing decidualized or non-decidualized hESCs in the presence of embryo conditioned medium (ECM) from high-quality embryos (fragmentation ≤ 20%) or from low-quality embryos (fragmentation > 20%) or in non-conditioned medium from the same dishes (control). ECM samples from 425 individually cultured human embryos were used in this study.

**Results:**

ECM from high-quality embryos, i.e., with a low percentage of fragmentation, actively stimulated decidualized hESC migration (*p* < 0.001). This effect was consistent throughout embryonic development from cleavage stage embryos with 2–7 cells (high quality vs. control; *p* = 0.036), 8–18 cells (high quality vs. control; *p* < 0.001) to morulae (high quality vs. control; *p* = 0.003). Additionally, linear regression analysis showed that hESC migration was influenced by embryo quality (fragmentation, β − 0.299; *p* = 0.025) and not developmental stage (cell number, β 0.177; *p* = 0.176) or maternal age (β − 0.036; *p* = 0.78). Opposite to decidualized hESCs, the migration response of non-decidualized hESCs was inhibited by ECM from high-quality embryos (*p* = 0.019). ECM from low-quality embryos, i.e., with a high percentage of fragmentation, did not cause an altered migration response in decidualized hESCs (*p* = 0.860) or non-decidualized hESCs (*p* = 0.986). Furthermore, ECM of both high- and low-quality human embryos did not influence the number of proliferating cells (*p* = 0.375) and the cell cycle time (*p* = 0.297) of non-decidualized or decidualized hESCs.

**Conclusion:**

This study reveals a mechanism by which high-quality human preimplantation embryos actively interact with the endometrium to increase their chances of successful implantation.

**Electronic supplementary material:**

The online version of this article (10.1007/s10815-017-1107-z) contains supplementary material, which is available to authorized users.

## Introduction

Implantation failure is still common in treatments for subfertility such as IVF/ICSI. More than half of all embryo transfers are not followed by a pregnancy resulting in live birth [1]. Consequently, improving the chances of implantation is considered an attractive strategy to improve ongoing pregnancy rates in IVF/ICSI [2].

For implantation to occur, a synchronous interplay has been suggested to take place between the embryo and the receptive endometrium [3]. However, little is known about the precise interaction between the embryo and the endometrium. An acknowledged predictor for successful implantation in IVF/ICSI is the morphology of the transferred embryo [4]. For each preimplantation embryo, a score can be assessed combining both its developmental stage and its morphological quality [5]. The developmental stage of the embryo is based on the consecutive cleavage cell stages, proceeding from the zygote and followed by the morula stage and multiple stages of blastulation. The morphological quality of the embryo can be determined by scoring blastomere size and symmetry and the percentage of fragmentation and multinucleation. However, in most embryo-grading systems, emphasis has been placed mainly on the percentage of fragmentation of blastomeres to determine embryo quality [5]. Fragmentation is defined as the presence of extracellular membrane-bound fragments that are inversely correlated with the developmental potential of the embryo [6]. Hence, fragmentation can be used as a parameter to determine the morphological quality of each embryo and thus its developmental potential. Subsequently, embryos can be ranked using both their developmental stage and their morphological quality. Transferring higher ranked embryos in IVF/ICSI results in higher live birth rates [7], however, whether higher ranked embryos achieve these improved live birth rates by actively interacting with the endometrium has not been elucidated.

Endometrial receptivity is acquired during the luteal phase of the menstrual cycle when the postovulatory progesterone rise induces decidualization of the human endometrial stromal cells (hESCs). Decidualization causes remodeling of non-decidualized hESCs into secretory decidualized hESCs that can facilitate implantation of an embryo [8].

In recent years, it has been shown that implantation can be studied in vitro by co-culturing hESCs with human preimplantation embryos or embryo-conditioned medium (ECM). Using these in vitro assays, it was shown that migration of hESCs is increased at implantation sites and that hESC migration is essential for trophoblast invasion to occur [9–11]. Moreover, it was shown that tripronuclear (3PN) embryos inhibited hESC migration and that co-culture of hESCs and arresting human embryos resulted in a downregulation of the secretion of endometrial interleukins [12, 13]. It has also been suggested that ECM from low-quality human embryos, estimated unsuitable for transfer in IVF/ICSI, induced a hypoxic stress response in hESCs which may negatively influence implantation [14].

The association between the morphology of a normally developing human embryo and hESC migration and proliferation has never been investigated. For this reason, it remains unclear whether a high-quality embryo regulates its implantation into the endometrium and how this implantation mechanism differs from low-quality embryos. In the present study, we systematically studied hESC migration and proliferation in response to ECM from human embryos of varying developmental stages and morphological quality.

## Materials and methods

### Ethical approval and informed consent

This study was approved by the Medical Review Ethics Committee of the VU University Medical Center. Written informed consent was obtained from all participating subjects that donated endometrial biopsies. ECM was collected from patients that did not object to storage and use of medical waste tissues.

### Endometrial biopsies and hESC isolation

hESCs were obtained from endometrial biopsies from hysterectomy specimens of patients that received surgery for benign indications (spotting due to a niche in the uterine cesarean scar *n* = 3 and dysmenorrhea e.c.i. *n* = 2). All included patients were premenopausal and had a history of proven fertility and at least one live birth. Patients received no hormonal treatment in at least 3 months prior to surgery. Patients with pathologies such as adenomyosis and endometriosis were excluded from this study. Endometrial biopsies were performed randomly in the menstrual cycle on hysterectomy specimens. Biopsied material was immediately suspended in pre-warmed Dulbecco’s Modified Eagle Medium/Nutrient Mixture F-12 phenol red-free medium (DMEM/F12; L-glutamine, HEPES; Thermo Fisher Scientific, Waltham, MA, USA) supplemented with 1% penicillin/streptomycin at 37 °C. The tissue was finely minced followed by enzymatic digestion in DMEM/F12 phenol red-free medium supplemented with > 125 U/ml collagenase IV and 1% penicillin/streptomycin. After complete suspension of the tissue fragments, collagenase activity was stopped by adding DMEM/F12 phenol red-free medium supplemented with 10% heat-inactivated fetal calf serum (FCS; Thermo Fisher Scientific, Waltham, MA, USA) and 1% penicillin/streptomycin (C-medium). Next, the cell suspension was filtered through a 76-μm sterile filter and pelleted by centrifugation at 750 g for 5 min. The cells were cultured in C-medium to enable attachment of the hESCs to the culture flask. After 3 h, the medium was replaced to selectively retain the attached hESCs. Purity of the isolated hESCs was assessed by positive immunostaining for vimentin (M0725, Agilent, CA, USA) and negative immunostaining for cytokeratin 18 (M7010, Agilent, CA, USA).

### hESC culture and decidualization

hESCs were cultured in C-medium at 37 °C, 5% C0_2_, and atmospheric oxygen concentrations. After reaching confluency, hESCs were passaged to start decidualization. In single wells of 48-well plates, 30,000 non-decidualized hESCs were seeded and decidualized for 5 consecutive days in DMEM/F12 phenol red-free medium supplemented with 10% heat-inactivated charcoal-stripped FCS (Thermo Fisher Scientific, Waltham, MA, USA), 0.5 mM 8-bromoadenosine 3′,5′-cyclic monophosphate (Sigma-Aldrich, Saint Louis, MO, USA), 1 μM medroxyprogesterone acetate (Sigma-Aldrich, Saint Louis, MO, USA), and 1% penicillin/streptomycin (D-medium). After six passages, new hESCs were isolated.

### Collection of embryo-conditioned medium

Embryo-conditioned medium (ECM) was collected from ICSI cycles. Routine ICSI procedures were followed, and all embryos were being handled according to local regulations and standard operating clinical procedures. From day 1 until day 3 after fertilization, embryos were cultured in Sage medium (Quinn’s advantage cleavage medium, Cooper Surgical, USA) supplemented with 5% human serum albumin (HSA; Vitrolife, Göteborg, Sweden), after which embryos were transferred to fresh Sage medium on day 3 (Quinn’s advantage blastocyst medium, Cooper Surgical, USA) supplemented with 5% HSA. Assessment of embryo morphology was performed daily. On day 4 after fertilization, when the embryos were removed to be used for cryopreservation, the empty culture dishes of assigned ICSI cycles were obtained. Individual culture droplets (20 μL) were collected in sterile 1.5 ml collection tubes. All samples were collected at day 4 after fertilization. Empty droplets from the same dishes, in which no embryo had been cultured, were stored as controls. Samples were snap-frozen in liquid nitrogen and stored at − 80 °C.

### Experimental groups of embryo-conditioned medium

ECM from five individually cultured embryos was pooled based on pre-defined categories of embryo quality and embryonic developmental stage. Low-quality embryos were defined by having more than 20% fragmentation, and high-quality embryos were defined by having equal to or less than 20% fragmentation.

### Migration assay

Either 30,000 decidualized or 30,000 non-decidualized hESCs were grown in confluent monolayers in single wells of a 48-well plate. To measure cell migration, a migration zone was created by scratching using a p200 pipet tip. After scratching, hESCs were washed once with PBS to remove unattached cells and 90 μL of pooled ECM was added directly onto monolayers of hESCs. To prevent evaporation, the cell cultures were overlaid with 150 μL of mineral oil (Irvine Scientific, Santa Ana, CA, USA). Directly after creation of the migration zone and after 18 h of co-culturing, phase contrast images were taken using a monochrome digital camera (Leica DFC365 FX) attached to an inverted microscope (Olympus IX71) with a U Plan FL 4x/0.13 PhL objective. To quantify the migration response of hESCs, the surface area of cells entering the migration zone was measured in pixels by using Image J software (version 1.50i). Surface reduction was determined by subtracting the surface area at 18 h from the surface area at 0 h.

### PKH26 and CSFE labeling

hESCs were grown until confluency and passaged. Decidualized hESCs were labeled with PKH26 dye (2 × 10^6 M in diluent C) for 5 min and non-decidualized hESCs were labeled with CSFE dye (0.5 μM in PBS) for 10 min at 37 °C and 5% C0_2_. Labeling was stopped by adding C-medium. Non-decidualized hESCs labeled with CSFE dye were pelleted by centrifugation at 750 g for 5 min and washed twice with C-medium. Decidualized hESCs labeled with PKH26 dye were pelleted by centrifugation at 400 g for 10 min and washed three times with C-medium. hESCs were seeded and assessed for viability 24 h later. Viable hESCs were passaged, and 15,000 decidualized hESCs (PKH26 labeled) and 15,000 non-decidualized hESCs (CSFE labeled) were mixed and seeded in single wells of a 48-well plate. After attachment of the hESCs, a migration zone was created and C-medium was replaced by control medium. Fluorescence and phase-contrast images were taken at 0 and 18 h after creation of the migration zone by using a monochrome digital camera (Leica DFC365 FX) attached to an inverted microscope (Olympus IX71) with a U Plan FL 4x/0.13 PhL objective.

### Proliferation assay and time-lapse microscopy

For proliferation assays, 30,000 non-decidualized hESCs were plated in single wells of a 48-well plate and cultured overnight before starting imaging using a Live-Cell Microscope II (DM8i) and N Plan Apo L 40x/0.55 Ph2 objective. Cultures were overlaid with mineral oil to prevent evaporation. Images were captured every 15 min for a total of 18 h (number of cell divisions) or 24 h (cell cycle time), at 37 °C and 5% C0_2_. Movies were manually analyzed for cell cycle time and total number of proliferating cells. The duration of a cell cycle was calculated by using the first time-point at which cell division occurred and the subsequent time-point at which the same cell underwent the next cell division.

### HaCaT cell line culture

HaCaT keratinocytes were cultured in Roswell Park Memorial Institute (RPMI) 1640 medium (Thermo Fisher Scientific, Waltham, MA, USA) supplemented with 8% FCS and 1% penicillin/streptomycin. After reaching confluency, HaCaT keratinocytes were passaged and 60,000 cells were seeded into individual wells of a 48-well plate to use for migration assays.

### Statistical analysis

Mean differences among experimental groups were tested by one-way ANOVA or Student’s *t* tests. To correct for multiple testing, we used Dunnett, Tukey, and Duncan post hoc tests when applicable. To statistically analyze the association between a continuous dependent variable and multiple independent variables, we used a linear regression analysis with migration as continuous dependent variable and maternal age, mean percentage of fragmentation, and mean number of blastomeres as independent continuous variables. Mean percentage of fragmentation and mean number of blastomeres were calculated from the matched values of the five individual embryos that were pooled per experiment. In all cases, IBM SPSS Statistics 23 software was used. *P* values < 0.05 were considered statistically significant.

## Results

### High-quality embryos stimulate migration of decidualized hESCs

**Fig. 1:**
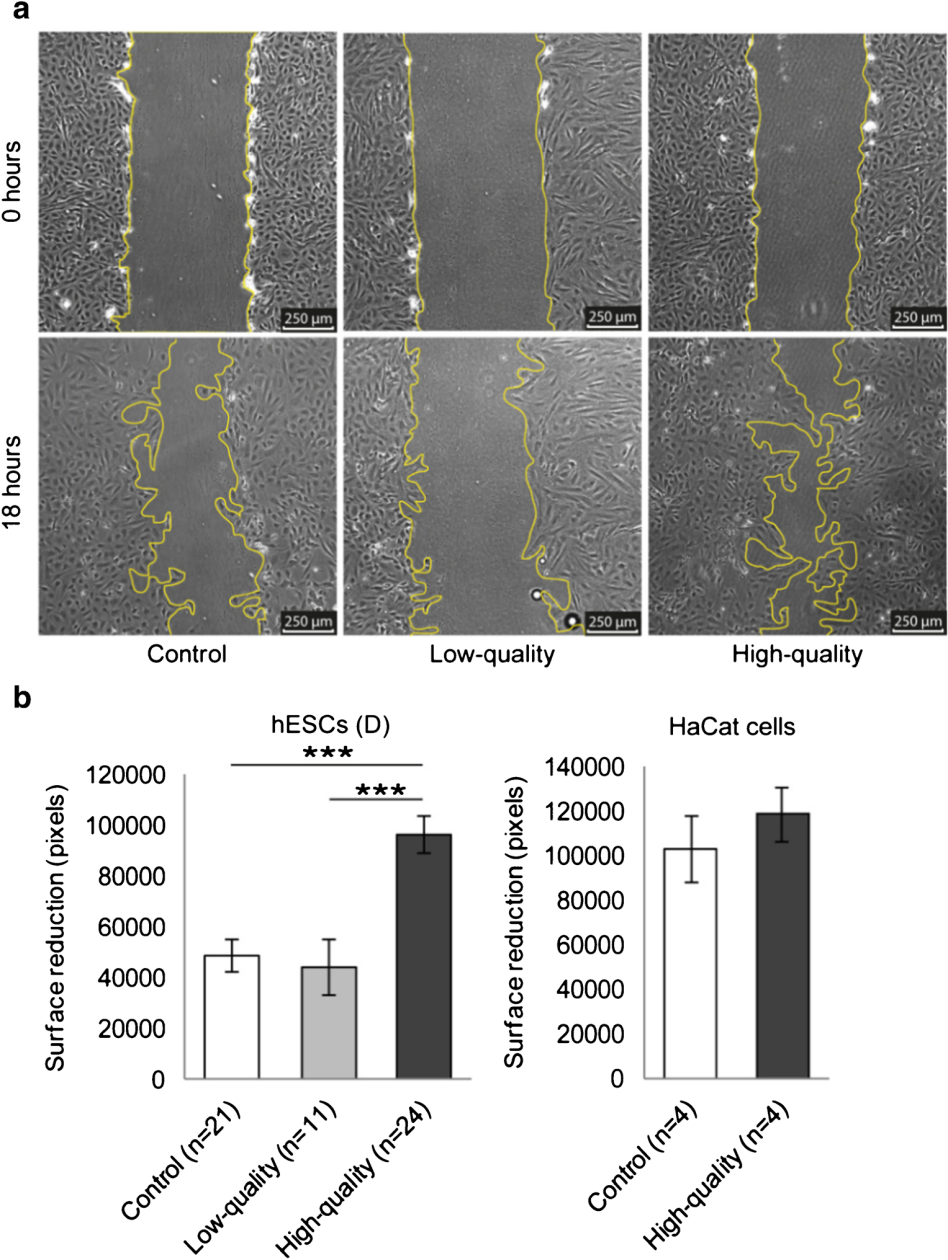
High-quality embryos stimulate migration of decidualized hESCs. **a** Images of the migration zone of decidualized hESCs co-cultured with ECM from low-quality embryos, high-quality embryos, or control taken at 0 and 18 h after creation of the migration zone. **b** Surface reduction of the migration zone is increased by co-culturing hESCs with ECM from high-quality embryos (*n* = 24, i.e., pooled ECM from 120 embryos) compared to control (*n* = 21, i.e., 105 pooled non-conditioned samples) or low-quality embryos (*n* = 11, i.e., pooled ECM from 55 embryos). **c** Surface reduction of the migration zone is not affected by co-culturing HaCaT cells with ECM from high-quality embryos (*n* = 4, i.e., pooled ECM from 20 embryos) or control (*n* = 4, i.e., 20 pooled non-conditioned samples). **b**, **c** Data are expressed as mean ± sem. Values that are significantly different are labeled by asterisks (***p < 0.001; one-way ANOVA). *hESCs* human endometrial stromal cells, *ECM* embryo-conditioned medium, *D* decidualized

For this study, we used ECM from a total of 425 individually cultured embryos. We first studied the combined effect of both the quality of the embryo, i.e., the percentage of fragmentation, and its developmental stage, i.e., the number of blastomeres, on decidualized hESC migration. Only hESC cultures with a purity of ≥ 98%, based on immunostainings for vimentin (stromal cell marker) and cytokeratin (epithelial cell marker), were used (Supplementary Fig. 1). Decidualization of hESCs was confirmed by expression of Prolactin (PRL) and Insulin-like growth factor-binding protein 1 (IGFBP1) by using Real-Time PCR (data not shown). Decidualized hESCs were incubated with ECM from low-quality embryos with 2–7 blastomeres (fragmentation > 20%; *n* = 11, i.e., pooled ECM from 55 embryos), high-quality embryos with eight or more blastomeres (fragmentation ≤ 20%; *n* = 24, i.e., pooled ECM from 120 embryos) or non-conditioned medium from the same dishes (control; *n* = 21, i.e., 105 pooled non-conditioned samples) and migration assays were performed. We observed a stimulation of decidualized hESC migration after co-culture with ECM from high-quality embryos (*p* < 0.001; Fig. 1a, b), whereas ECM from low-quality embryos had no effect (*p* = 0.860; Fig. 1a, b). To control whether the observed migration response was specific to hESCs or might occur in various human cell types, we repeated the migration assay using a keratinocyte cell line (HaCaT) instead of hESCs. HaCaT cells were co-cultured with pooled ECM from high-quality embryos (eight or more blastomeres; fragmentation ≤ 20%; *n* = 4, i.e., pooled ECM from 20 embryos) or control (*n* = 4, i.e., 20 pooled non-conditioned samples). We did not observe any statistically significant difference in migration of HaCaT cells after co-culture with ECM from high-quality embryos compared to control (*p* = 0.432; Fig. 1c).

### High-quality embryos from all developmental stages stimulate migration of decidualized hESCs

**Fig. 2:**
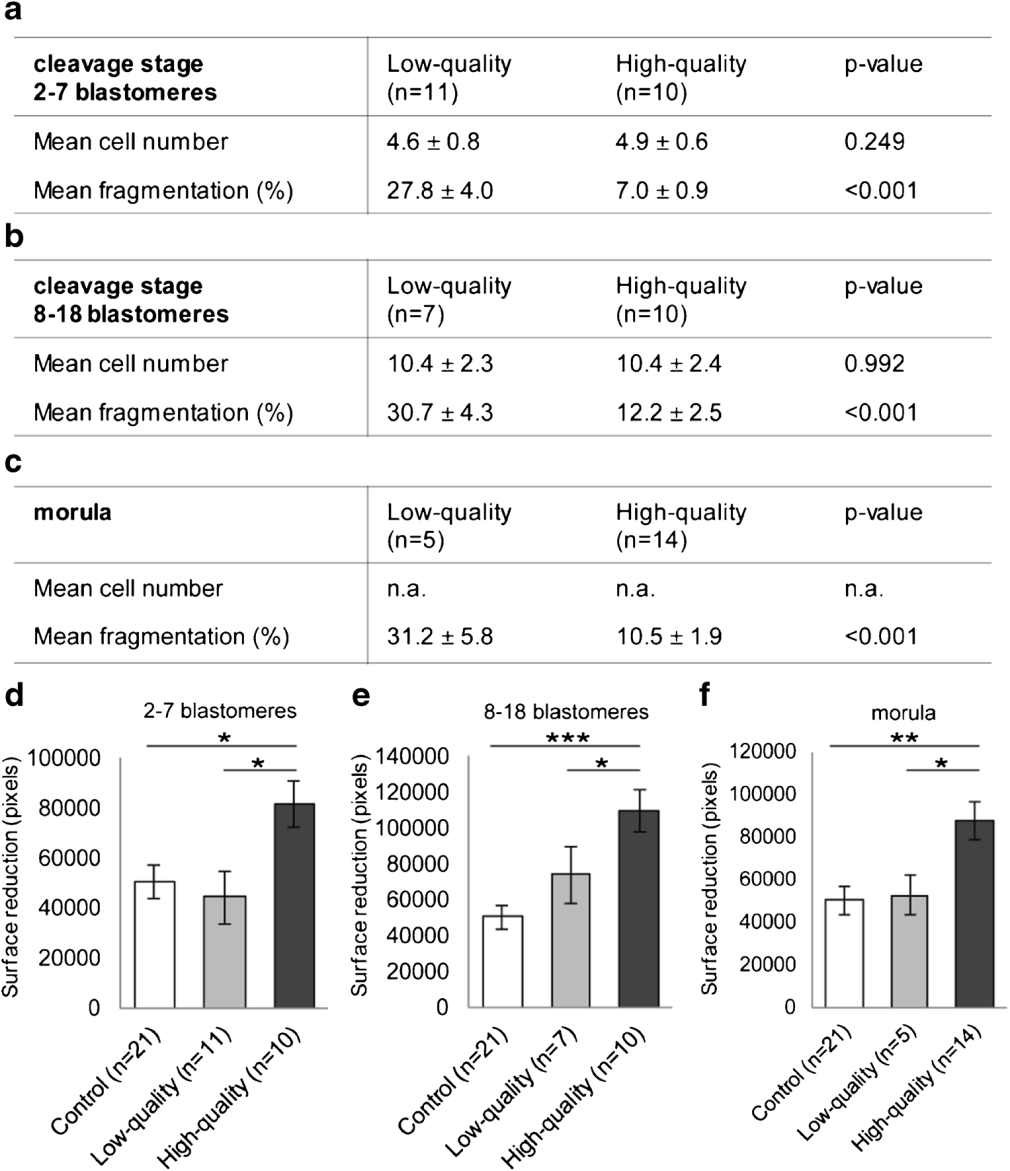
High-quality embryos from all developmental stages stimulate migration of decidualized hESCs. **a**, **b**, **c** Samples used from cleavage stage embryos with 2–7 cells (**a**), cleavage stage embryos with 8–18 cells (**b**) or morulae (**c**). The mean percentage of fragmentation was significantly different between samples, but the mean cell number was not significantly different. **d**, **e**, **f** Surface reduction of the migration zone is increased by co-culturing hESCs with ECM from high-quality embryos compared to control or low-quality embryos at the cleavage stage with 2–7 blastomeres (**d**), the cleavage stage with 8–18 blastomeres (**e**), and the morula stage (**f**). **a**–**f** Each experiment (*n*) was performed with pooled ECM from five individually cultured embryos. **d**, **e**, **f** Data are expressed as mean ± sem. Values that are significantly different are labeled by asterisks (***p < 0.001, **p < 0.01, *p < 0.05; one-way ANOVA). *ECM* embryo-conditioned medium

**Fig. 3:**
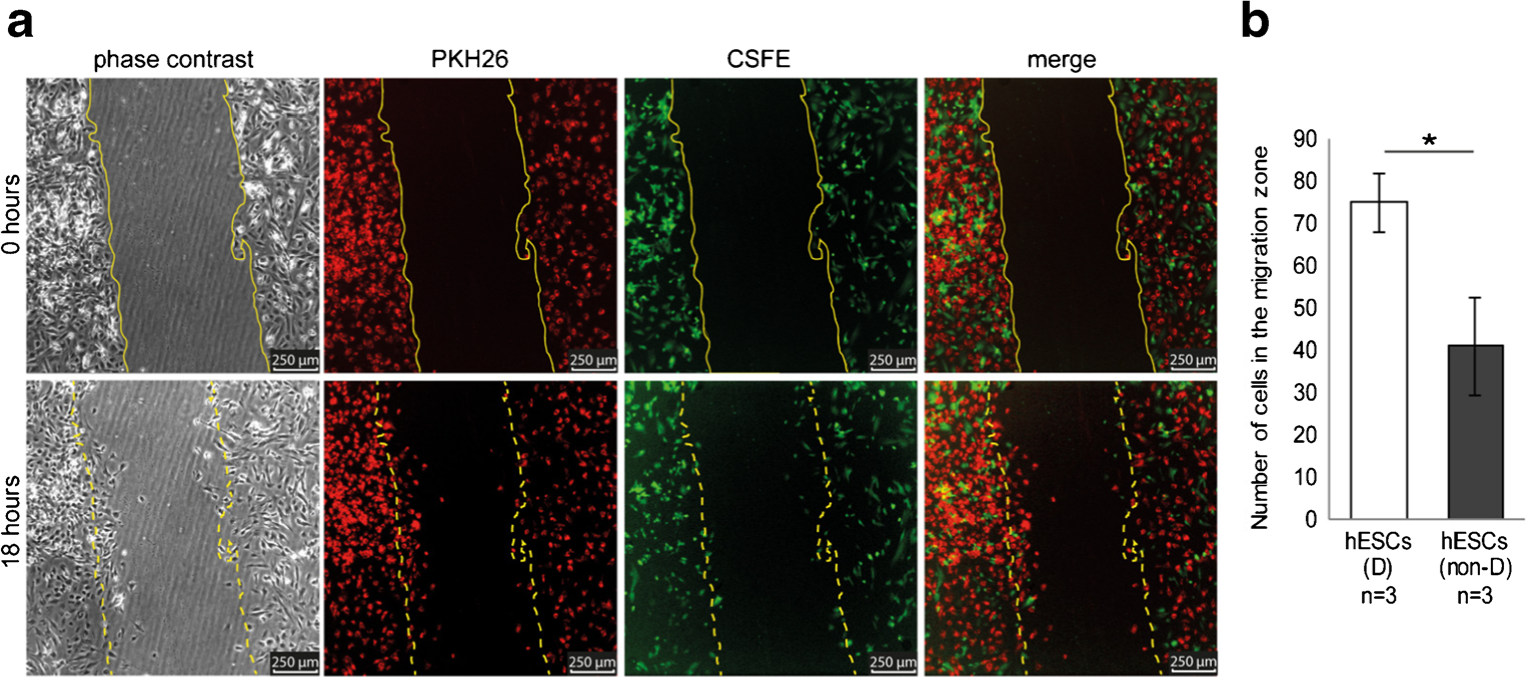
Decidualized hESCs migrate more actively compared to non-decidualized hESCs. **a** Microscopy images taken at 0 or 18 h after creation of the migration zone. Decidualized hESCs were labeled with PKH26 (red), and non-decidualized hESCs were labeled with CSFE (green). **b** The number of decidualized hESCs that have migrated into the migration zone is significantly higher than the number of non-decidualized hESCs. **b** Data are expressed as mean ± sem. Values that are significantly different are labeled by asterisks (*p < 0.05; Student’s *t* test). *hESCs* human endometrial stromal cells, *D* decidualized, *non-D* non-decidualized

To unravel whether the observed stimulation of hESC migration was influenced by either the quality of the embryo, i.e., the percentage of fragmentation, or its developmental stage, i.e., the number of blastomeres, we co-cultured decidualized hESCs with ECM from embryos matched on developmental stage but with different quality. We used ECM from cleavage stage embryos containing 2–7 blastomeres of both low-quality (*n* = 11, i.e., pooled ECM from 55 embryos) and high quality (*n* = 10, i.e., pooled ECM from 50 embryos), cleavage stage embryos containing 8–18 blastomeres of both low-quality (*n* = 7, i.e., pooled ECM from 35 embryos) and high-quality (*n* = 10, i.e., pooled ECM from 50 embryos) and morulae of both low-quality (*n* = 5, i.e., pooled ECM from 25 embryos) and high-quality (*n* = 14, i.e., pooled ECM from 70 embryos). The mean percentage of fragmentation was significantly different between low-quality and high-quality embryos within each experimental group (all comparisons *p* < 0.001; Fig. 2a, b, c). The mean number of blastomeres was not significantly different between low-quality or high-quality embryos within the groups of cleavage stage embryos containing 2–7 blastomeres (*p* = 0.249; Fig. 2a) or 8–18 blastomeres (*p* = 0.992; Fig. 2b). We found stimulation of decidualized hESC migration after co-culture with ECM from high-quality embryos compared to control at all developmental stages: cleavage stage embryos with 2–7 blastomeres (*p* = 0.036; Fig. 2d), cleavage stage embryos with 8–18 blastomeres (*p* < 0.001; Fig. 2e), and morulae (*p* = 0.003; Fig. 2f). There was no effect of ECM from low-quality embryos on the migration response of decidualized hESCs compared to control at all developmental stages: cleavage stage embryos with 2–7 blastomeres (*p* = 0.901; Fig. 3d), cleavage stage embryos with 8–18 blastomeres (*p* = 0.240; Fig. 2e), and morulae (*p* = 0.972; Fig. 2f). To confirm that indeed embryo quality influenced hESC migration, we performed a multivariate linear regression analysis. We found a significant correlation between decidualized hESC migration and the mean percentage of fragmentation (β − 0.299; *p* = 0.025) but not the number of blastomeres (β 0.177; *p* = 0.176) or maternal age (β − 0.036; *p* = 0.780). The stimulatory effect on decidualized hESC migration was thus specifically caused by all high-quality embryos, i.e., having a low percentage of fragmentation.

### Decidualized hESCs migrate more actively compared to non-decidualized hESCs

To investigate whether the basic migration response of hESCs changed upon decidualization, we studied cell migration of a mixed cell population containing both decidualized and non-decidualized hESCs. Decidualized hESCs were labeled with the red fluorescent dye PKH26, and non-decidualized hESCs were labeled with the green fluorescent dye CSFE. Fluorescence microscopy showed that significantly more decidualized hESCs migrated into the migration zone compared to non-decidualized hESCs (75 ± 7.0 SD versus 41 ± 12 SD, *p* = 0.013; Fig. 3a, b).

### High-quality embryos inhibit migration of non-decidualized hESCs

**Fig. 4:**
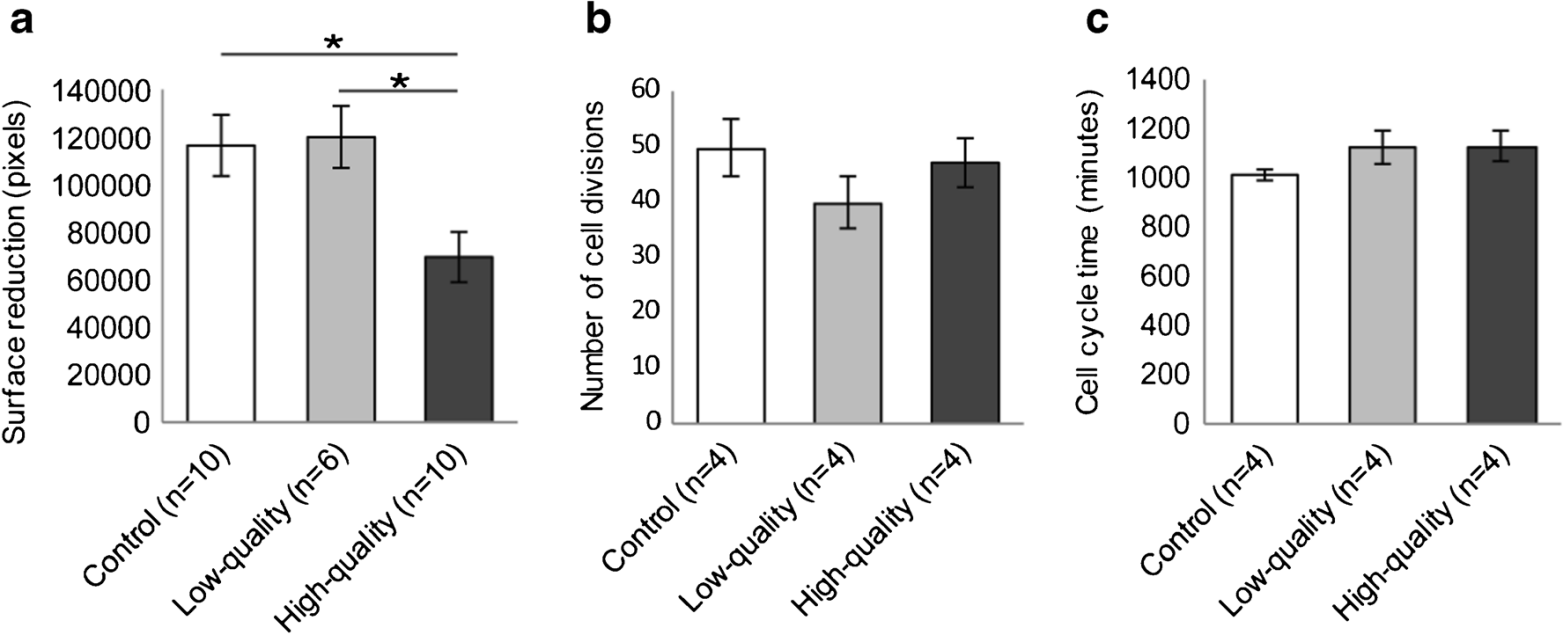
High-quality embryos inhibit migration of non-decidualized hESCs. **a** Surface reduction of the migration zone is decreased after co-culturing non-decidualized hESCs with ECM from high-quality embryos (*n* = 10, i.e., pooled ECM from 50 embryos) compared to control (*n* = 10, i.e., 50 pooled non-conditioned samples) or low-quality embryos (*n* = 6, i.e., pooled ECM from 30 embryos). **b**, **c** The number of cell divisions (**b**) or the cell cycle time (**c**) of non-decidualized hESCs is not different after co-culture with ECM from high-quality embryos (*n* = 4, i.e., pooled ECM from 20 embryos) compared to control (*n* = 4, i.e., 20 pooled non-conditioned samples) or low-quality embryos (*n* = 4, i.e., pooled ECM from 20 embryos). **a**, **b**, **c** Data are expressed as mean ± sem. Values that are significantly different are labeled by asterisks (*p < 0.05; one-way ANOVA). *ECM* embryo-conditioned medium

Next, the migration response of non-decidualized hESCs was tested in response to ECM from either low-quality or high-quality embryos. Non-decidualized hESCs were incubated with pooled ECM from low-quality embryos containing 2–7 blastomeres (fragmentation > 20%; *n* = 6, i.e., pooled ECM from 30 embryos), high-quality embryos containing eight or more blastomeres (fragmentation ≤ 20%; *n* = 10, i.e., pooled ECM from 50 embryos) or control (*n* = 10, i.e., 50 pooled non-conditioned samples), and incubated for 18 h. In contrast to decidualized hESCs, the migration response of non-decidualized hESCs was inhibited by ECM from high-quality embryos (*p* = 0.019; Fig. 4a), whereas ECM from low-quality embryos had no effect (*p* = 0.986; Fig. 4a). Because, in contrast to decidualized cells, non-decidualized hESCs are highly proliferative, we continued to investigate whether the inhibition of migration could be caused by changes in proliferation. Using time-lapse microscopy, we monitored non-decidualized hESCs during co-culture with pooled ECM from low-quality embryos containing 2–7 blastomeres (fragmentation > 20%; *n* = 4, i.e., pooled ECM from 20 embryos), high-quality embryos containing eight or more blastomeres (fragmentation ≤ 20%; *n* = 4, i.e., pooled ECM from 20 embryos) or control (*n* = 4, i.e., 20 pooled non-conditioned samples). During 18 h of co-culturing, we assessed both the total number of proliferating cells and the cell cycle time. ECM from neither high- nor low-quality human embryos influenced the total number of proliferating cells (*p* = 0.375; Fig. 4b) or the cell cycle time of non-decidualized hESCs compared to control (*p* = 0.297; Fig. 4c). High-quality preimplantation embryos thus stimulated migration of decidualized hESCs and suppressed migration of non-decidualized hESCs.

## Discussion

Pregnancy rates in IVF/ICSI increase by transferring embryos with higher morphological quality [15]. Current hypotheses of successful implantation underscore the importance of crosstalk between the receptive endometrium and a high-quality embryo [16, 17]. This study demonstrates a mechanism by which normally developing high-quality embryos interact with the receptive endometrium to increase their chance of successful implantation.

Our study demonstrates an increased migration response of decidualized hESCs triggered by ECM from high-quality human embryos, i.e., with a low percentage of fragmentation, irrespective of their developmental stage. Concurrent with decreased invasiveness of non-decidualized hESCs [18], our study also demonstrates that non-decidualized hESCs have a lower migratory capacity than decidualized hESCs. Moreover, we found a further reduction of non-decidualized hESCs migration after co-culture with ECM from high-quality embryos. High-quality embryos thus have opposing effects on decidualized and non-decidualized hESCs. On the other hand, low-quality embryos, i.e., with a high percentage of fragmentation, did not influence the migration response of either decidualized or non-decidualized hESCs. Hence, we show that embryo quality is paramount to determine hESC migration and thus the implantation response of the endometrium.

Recent evidence indicated that low-quality human embryos inhibited migration of decidualized hESCs during implantation, whereas no effect of high-quality embryos was described [19]. In the present study, we found no effect of low-quality embryos on hESC migration but did find an effect of high-quality embryos. The following methodological differences may explain this discrepancy. First, the definition of high- and low-quality embryos differs between various studies. The only study that investigated the effect of embryo quality on hESC migration defined low-quality as the presence of three pronuclei, while morphological quality was not investigated [12]. However, embryos with three pronuclei can develop normally with good morphological quality and are able to implant. Therefore, these embryos may not truly reflect low quality in regard to their implantation potential. In contrast, by using ECM, in our study, we were able to adhere to pre-defined embryo groups based on both developmental stage and the percentage of fragmentation, in agreement with scoring and selection procedures of high-quality human embryos in IVF-laboratories worldwide.

**Fig. 5:**
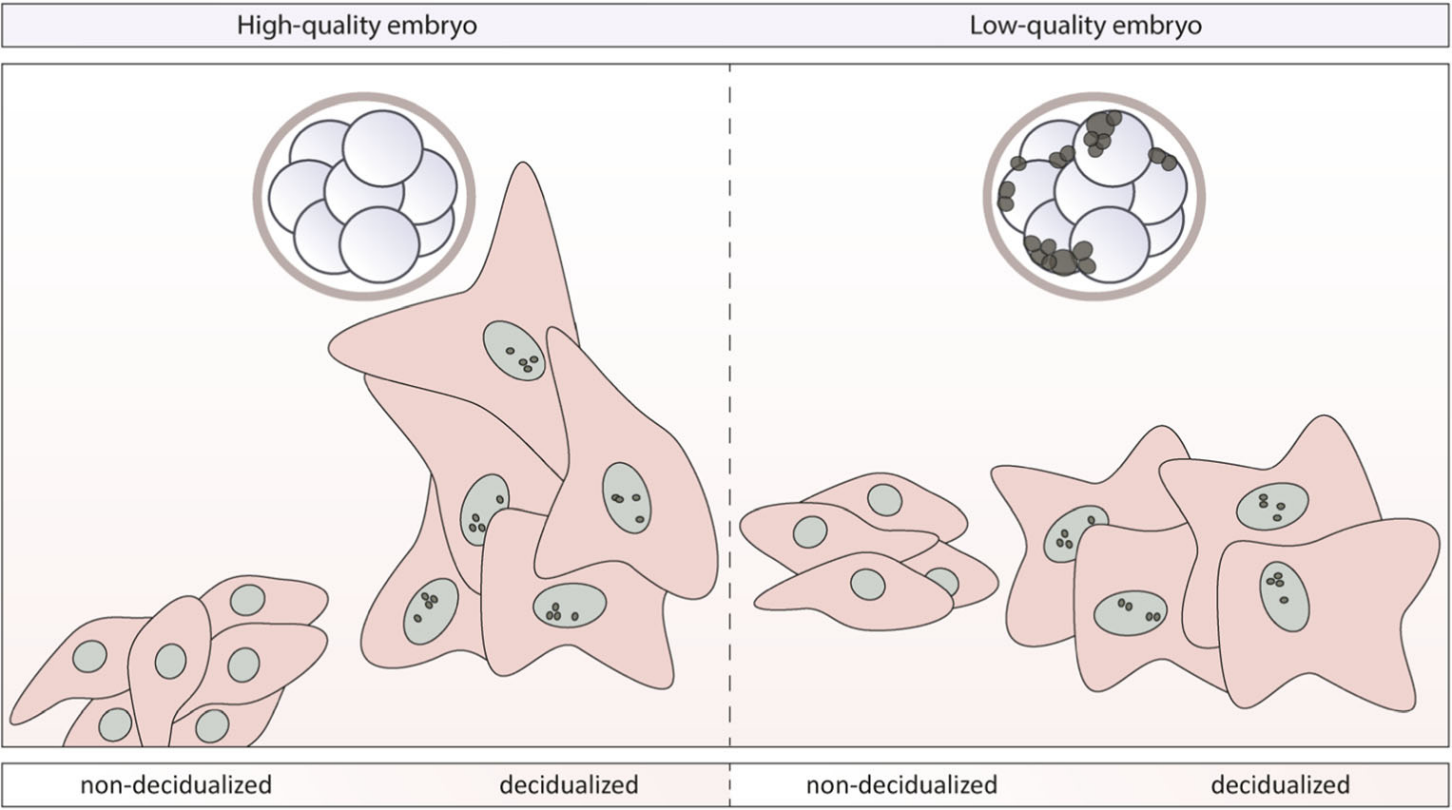
Paracrine signaling of the preimplantation embryo depends on embryo quality and differently regulates migration of decidualized or non-decidualized hESCs. High-quality human preimplantation embryos (fragmentation ≤ 20%) stimulate migration of decidualized hESCs while repressing migration of non-decidualized hESCs (left). Low-quality human preimplantation embryos have no effect on the migration of decidualized hESCs or non-decidualized hESCs (right)

In summary, our study shows that the human preimplantation embryo interacts with its surroundings during implantation (Fig. 5). Paracrine signaling of high-quality embryos, i.e., with a low percentage of fragmentation, actively stimulated migration of decidualized hESCs, whereas non-decidualized hESCs were actively being suppressed by signals from the same high-quality embryos. This indicates a shift in the responsiveness of hESCs to embryonic cues. Moreover, hESCs seem able to sense the quality of human embryos and respond accordingly to increase the chances of successful implantation. This effect was consistent throughout embryonic development, from cleavage stage embryos to morulae, and was not found in lower quality embryos.

Although the nature of these embryonic paracrine signals and their downstream molecular pathways in hESCs needs to be further investigated, this study contributes to our understanding of human implantation, with the ultimate aim to develop more effective treatment strategies for subfertility.

## Electronic supplementary material

Supplementary Figure 1 (DOCX 518 kb)
