# Supplementary material for: High-quality human preimplantation embryos actively influence endometrial stromal cell migration
Source: J Assist Reprod Genet. 2017 Dec 28;35(4):659–67. doi: 10.1007/s10815-017-1107-z (PMC5949101; doi:10.1007/s10815-017-1107-z)
Supplement: Supplementary file 1 — (DOCX 518 kb) [file 10815_2017_1107_MOESM1_ESM.docx]

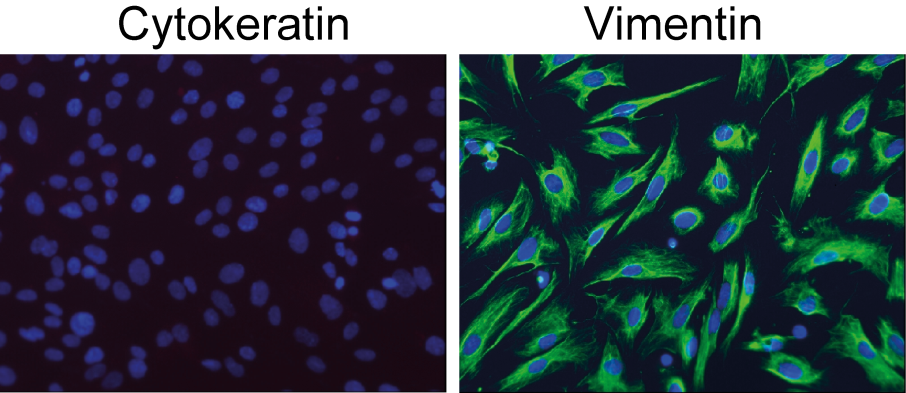


**Supplementary Figure 1**

Human endometrial stromal cells are negative for cytokeratin (red; left) and positive for vimentin (green; right). Nuclei are stained with DAPI (blue). Only hESCs cultures with a purity of ≥98% were used for subsequent experiments.
